# The lingering mysteries of metastatic recurrence in breast cancer

**DOI:** 10.1038/s41416-020-01161-4

**Published:** 2020-11-26

**Authors:** Alessandra I. Riggio, Katherine E. Varley, Alana L. Welm

**Affiliations:** Department of Oncological Sciences, Huntsman Cancer Institute, University of Utah, Salt Lake City, UT USA

**Keywords:** Breast cancer, Metastasis

## Abstract

Despite being the hallmark of cancer that is responsible for the highest number of deaths, very little is known about the biology of metastasis. Metastatic disease typically manifests after a protracted period of undetectable disease following surgery or systemic therapy, owing to relapse or recurrence. In the case of breast cancer, metastatic relapse can occur months to decades after initial diagnosis and treatment. In this review, we provide an overview of the known key factors that influence metastatic recurrence, with the goal of highlighting the critical unanswered questions that still need to be addressed to make a difference in the mortality of breast cancer patients.

## Background

**Fig. 1:**
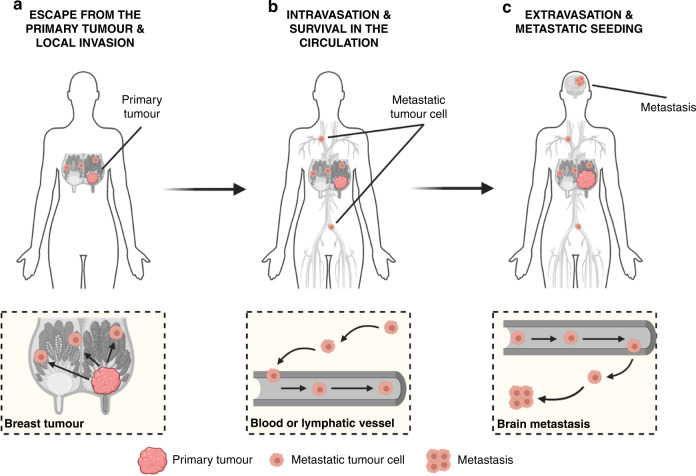
The metastatic cascade as a multistep process. **a** Escape of tumour cells from the primary site and local invasion of surrounding tissues. Breast cancer is depicted here as the primary tumour. **b** Intravasation and survival of metastatic tumour cells into the circulation. Please note that this process can occur via both the haematogenous and lymphatic systems. **c** Extravasation of tumour cells from the circulation and metastatic seeding at distant sites. While dissemination of breast tumour cells can occur to the bones, lungs and liver, the brain is depicted here as the deadliest metastatic site for breast cancer patients. Figure created with BioRender.com.

With a total of 18,989,634 new cases and 10,052,507 associated deaths estimated to occur in 2020,^1^ cancer remains a major health problem worldwide.^2^ Metastasis is the dissemination of tumour cells from the primary neoplasm to secondary sites in a multistep process that is often depicted as a simple series of sequential events:^3,4^ escape from the primary tumour and local invasion, intravasation and survival in the circulation and extravasation and metastatic seeding (Fig. 1). This process is responsible for >90% of tumour-related deaths, often due to the impairment of vital organ function.^5,6^ Metastatic disease can occur de novo, in which metastases are present at the original diagnosis, the cancer having already spread prior to detection. However, it is most often the result of relapse (recurrence), where metastases manifest after definitive treatment.^7^ Metastatic recurrence is a significant problem in patients with breast cancer, the most frequently diagnosed malignancy and the second leading cause of cancer-related death among women worldwide.^2^ Although the incidence of distant relapse has been shown to be decreasing and survival times for patients with recurrent disease have improved,^8^ 20–30% of patients with early breast cancer still die of metastatic disease.^9,10^ In line with clinical observations that distinct organ tropisms are displayed by different tumour types,^11,12^ dissemination of breast cancer cells has predominantly been reported to the bones, lungs, liver and brain, in addition to lymph nodes.^13^ Despite the bone being the most and the brain being the least-affected metastatic sites,^14^ the median survival of breast cancer patients ranges from 36.0 to 8.0 months, depending on the presence of bone versus brain metastases, respectively.^15^ Protracted intervals between diagnosis and recurrence have been proposed to be the result of tumour dormancy, whereby clinically undetectable minimal residual disease (MRD) can lie asymptomatic for many years to decades.^16^ This phenomenon appears to reflect not only the metastatic behaviour of cancer cells that are able to escape from the primary tumour and spawn multiple distant metastases prior to diagnosis, but also their ability to remain ‘dormant' in secondary sites and thereby resistant to anti-proliferative agents.^17^ Thus, given its systemic nature and inevitable resistance to therapy, metastatic recurrence is largely incurable and remains the foremost concern for cancer patients and their caregivers. Despite the urgency, however, an understanding of the biological underpinnings of relapse is still lacking.^18^ This review focuses on the questions surrounding metastatic relapse, using breast cancer as a key example, given its wide window of recurrence spanning from months to decades from initial treatment. This range in recurrence intervals is likely to reflect, at least in part, tumour cell dissemination, the balance between cell-extrinsic and cell-intrinsic factors in the metastatic environment and the putative dormancy of metastatic cells at distant sites. We also discuss the implications of new approaches that are capable of earlier detection of recurrence and speculate on new strategies that might be employed to target MRD.

## The varied and unpredictable nature of metastatic recurrence in breast cancer

### Breast cancer is a heterogeneous disease

Based on the presence or absence of the oestrogen receptor (ER) and progesterone receptor (PR), and the expression and amplification of the human epidermal growth factor receptor type 2 (HER2), breast cancer can be divided into three clinical subtypes: hormone-receptor (HR)-positive (HR^+^; ER^+^, PR^+/–^ and HER2^–^), HER2-positive (HER2^+^) and triple-negative (TN; ER^–^, PR^–^ and HER2^–^).^9,19^ In the United States, 71% of breast cancers are HR^+^, 17% are HER2^+^ and 12% are TN.^20^ Following the discovery of five intrinsic molecular subgroups of the disease based on a 50-gene expression classifier (PAM50)—luminal A, luminal B, HER2-enriched, basal-like and normal-like—it became apparent that a large degree of unappreciated molecular heterogeneity exists across and within each subtype of breast cancer.^21^ While TN and HER2^+^ patients often present with basal-like and HER2-enriched cancers, respectively, HR^+^ women are usually diagnosed with luminal A or luminal B tumours. However, despite sharing some common traits, luminal A cancers (HER2^–^) are generally ER^+^, PR high and Ki67 low, resulting in low-grade, slow-proliferating neoplasms, whereas luminal B tumours (HER2^–^ or HER2^+^) are typically ER^+^, PR variable and Ki67 variable, translating into more aggressive cancers with a higher proliferative rate.^22^

### The wide window of relapse in breast cancer

In contrast with other solid tumours in which metastatic recurrence can occur within a few weeks (in the case of lung cancer) or a few years (in the case of colorectal cancer) following diagnosis,^23^ breast cancer is characterised by a wide window of relapse, spanning months to decades after surgery.^24^ The basis of this peculiar pattern of recurrence is still elusive, but is likely to be linked to the aforementioned molecular differences underlying each subgroup, with basal-like and HER2-enriched patients experiencing early relapses (within the first 5 years after diagnosis), as opposed to patients with luminal cancers characterised by a more favourable prognosis.^25–27^ Nonetheless, patients with luminal B tumours tend to have shorter survival times than luminal A patients.^28^ In addition to the contribution of the molecular subtype of the primary tumour, the risk and timing of recurrence is also influenced by other tumour-related factors that constitute the pillars of the TNM (tumour-node-metastasis) classification system: tumour size and spread (T), regional lymph node involvement (N) and the presence of distant metastasis (M).^29^ Based on the premise that the chance of survival is intimately linked to the anatomic extent (i.e., stage) of the disease, the TNM staging system stratifies cancer patients at diagnosis into four stages—with patients with Stage I disease having a much better prognosis as opposed to patients with Stage IV disease—thus representing the gold standard tool for prognostication.^30^

### The black box of recurrence

Given the above-mentioned features of their primary breast cancer, population-based statistics and, sometimes, primary tumour gene expression tests,^31^ newly diagnosed patients receive a prognosis for their risk of recurrence. However, the heterogeneity of breast cancer is such that accurate predictions cannot be made, which generally leads to situations where the disease is overtreated.^32^ Furthermore, many breast cancer patients, especially those with HR^+^ cancers who typically experience late relapse as described above, never know if they are ‘in the clear’ following treatment of their primary tumour, or whether they are still at risk of relapse. As breast cancer remains the most frequently diagnosed malignancy among women,^2^ identifying the drivers (and predictors) of recurrence in these patients is of paramount importance. To do so, several questions remain to be addressed. Which tumour cells are responsible for metastatic recurrence? What dictates successful versus unsuccessful metastasis? Is tumour dormancy the sole explanation for relapse and, if so, can it be targeted to prevent recurrence? Importantly, can earlier detection of recurrence improve breast cancer outcomes? In the following sections, we discuss these questions, with the ultimate goal of highlighting areas that are still in urgent need of research in order to solve the mystery of metastatic relapse. We focus especially on HR^+^ breast cancer, as this subtype kills more patients than all other breast cancer subtypes, yet receives little recognition for its potential for metastatic relapse due to long disease-free intervals.

## Disseminated tumour cells as culprits for metastatic recurrence

**Fig. 2:**
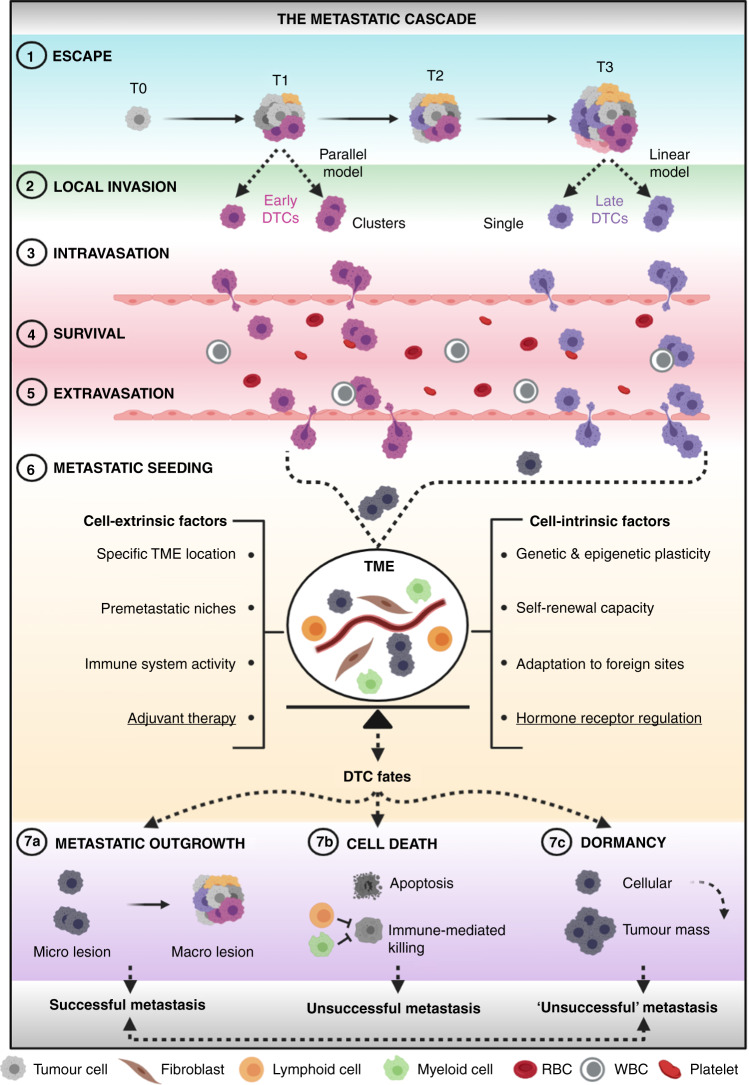
Tumour cell dissemination: the route to metastatic success or failure. (1) Escape of DTCs at early (parallel model) or late (linear model) stages of tumorigenesis, either as single cells or clusters. (2) Local invasion of nearby host tissues. (3) Intravasation into the circulation. (4) Survival in the circulation. (5) Extravasation from the circulation into distant sites. (6) Metastatic seeding at distant loci, wherein crosstalk between DTCs and host cells of the TME takes place (DTCs are now depicted in grey as the fate of early and late tumour cells at this stage is unclear). This is the most critical step of the metastatic cascade in which a fine balance between cell-extrinsic and cell-intrinsic factors seems to dictate DTC fate. Underlined are adjuvant therapy and hormone-receptor regulation as two key cell-extrinsic and cell-intrinsic determinants, respectively, of metastatic success or failure in HR^+^ breast cancer. (7a) metastatic outgrowth, (7b) cell death and (7c) dormancy are three distinct fates that DTCs can undergo through various mechanisms. Whilst cell death is irreversible, ‘unsuccessful’ metastases composed of dormant cancer cells might eventually resume their growth and give rise to deadly lesions. DTCs disseminated tumour cells, RBC red blood cell, T tumour stage, TME tumour microenvironment, macro macroscopic, micro microscopic, WBC white blood cell. Figure created with BioRender.com.

Metastatic relapse is attributed to the outgrowth of cancer cells that have escaped from the primary tumour and take up residence in secondary sites. Cancer cells that physically detach from a primary source and seed distant sites are known as disseminated tumour cells (DTCs).^33^ The process whereby DTCs transform a localised cancer into a systemic disease is called the metastatic cascade^34^ (Fig. 2). In the next few sections, the seven key steps comprising this complex biological process are discussed with the goal to shed light on the ‘when’ and ‘how’ of DTC dissemination. Importantly, while depicting the metastatic cascade as an orderly series of sequential events—starting from the primary tumour and ending in a distant metastatic site—it should be noted that DTC spread can take place through multiple routes and different directions.^35^ Accordingly, clinical evidence of self-seeding^36^—whereby a metastatic cell re-infiltrates its primary tumour—and of metastasis-to-metastasis spread^37,38^ has been documented, with one such study in HR^+^ breast cancer patients reporting a common origin between lymph node and distant metastases in up to 25% of cases.^39^

### When do tumour cells disseminate? Early versus late dissemination

The first step of the metastatic cascade refers to the ability of cancer cells to escape from the primary tumour. In regard to the ‘when’, two different models of metastatic dissemination—linear and parallel—have been proposed^33^ (Fig. 2.1). The linear model, based on the natural stepwise progression of cancer^40^ and on the aforementioned correlation between primary tumour size and the risk of relapse,^30^ surmises that only advanced neoplasms contain enough molecular aberrations to facilitate all of the obligate steps of the metastatic cascade. Accordingly, only fully malignant cancer cells (termed ‘late’ DTCs) can break away from the primary source and give rise to deadly metastases.^41^ The parallel model, by contrast, posits that metastatic dissemination can be a precocious event in tumorigenesis in which incipient tumour cells (termed ‘early’ DTCs) can develop into deadly metastases in parallel with each other and the primary tumour. Accordingly, early DTCs would simultaneously, yet independently, acquire genetic and epigenetic alterations as compared with their tumour of origin, perhaps based on the specific niche-related microenvironments they lodge in ref. ^33^. This model is supported by a plethora of tumour-growth rate studies asserting that some metastases were too big to have been initiated by late-stage tumours,^42,43^ as well as by the detection of secondary lesions in patients with cancers of unknown origin.^33^ Furthermore, DTCs have been detected in the bone marrow of patients with early-stage cancer,^44–49^ wherein their presence did not correlate with tumour stage, subtype, histological grade nor axillary lymph node status.^50^ Altogether, these results suggest that the parallel model of cancer cell dissemination is a much more common phenomenon than previously thought (Fig. 2.1). Although more compelling data are needed to elucidate the temporal pattern of DTC spread, the majority of metastatic human neoplasms appear to have already disseminated—albeit without being detected—at the time of diagnosis,^51^ despite the fact that metastases tend to be associated with larger tumours (as mentioned above^30^). Thus, both parallel and linear dissemination might occur, even within the same patient. Importantly, despite the fact that both models assume a clonal relationship between primary tumours and metastases, they differ not only in regard to the timing of the emergence of metastasis-prone DTCs (as discussed above), but also in the expected genetic divergence between paired sites—bigger in the case of parallel dissemination, smaller in the case of linear progression.^35^ Consequently, phenotypic differences that distinguish early and late DTC functions have been reported,^45,46,52^ the implications of which are discussed later in the text.

### How do tumour cells disseminate?

The second step of the metastatic cascade refers to DTC ability to invade adjacent local tissues (Fig. 2.2). During this step, which occurs through degradation of the basement membrane and remodelling of the extracellular matrix (ECM),^53^ DTCs can disseminate either as single cells or as clusters of cells.^54^ At the basis of these two interconverting mechanisms of invasion—amoeboid and mesenchymal migration on one side, and collective migration on the other^55^—there seems to be DTC capability to acquire transitional stages between the epithelial and mesenchymal phenotypes.^18,56^ The third step of the cascade is known as intravasation^6^ (Fig. 2.3)—the entry of DTCs into the lumina of blood vessels (most common) or lymphatic vessels—a process that appears to be partly mediated via a mitosis-induced mechanism elicited by cancer cells located along the vessel periphery.^57^ Once in the circulation, a plethora of challenges are faced by DTCs—at this point referred to as circulating tumour cells (CTCs)^58^—first and foremost their ability to survive a variety of stresses, such as avoiding anoikis (a form of apoptosis due to loss of integrin-dependent anchorage to the ECM), evading the immune system and overcoming haemodynamic shear forces^4^ (Fig. 2.4). One of the ways this is thought to occur is through the formation of circulating CTC clusters comprising other cancer^54^ and/or immune^59,60^ cells. Despite being rarer than single CTCs, CTC clusters were shown to have increased metastatic potential as compared with their single cell counterparts.^61^ Extravasation, the arrest of CTCs at distant sites and their exit from the circulation, represents the fifth step of the metastatic cascade (Fig. 2.5). The previously discussed metastatic tropism—the ability of CTCs to actively home to specific organs^12^—and the passive physical trapping of cancer cells due to the vasculature layout have been proposed as putative mechanisms driving such process.^6^ At this stage, as well as for any of the aforementioned steps involving cancer cell passage through physical constraints, factors such as reduced actin cytoskeleton anisotropy, cell stiffness and focal adhesion density—all linked to increased MAPK signalling—have been reported to be key determinants of efficient DTC spread.^62^ The sixth step of the cascade is metastatic seeding, a process that results in the docking of extravasated DTCs in distant foreign sites (Fig. 2.6). As discussed in the sections below, this is perhaps the most crucial step of the whole cascade,^63^ as it challenges DTC potential and shapes DTC fate in such a way that might determine whether successful or unsuccessful metastases are formed. In this regard, findings from breast cancer mouse models have suggested that early DTCs might be more capable of metastatic seeding, yet less competent at forming primary tumours upon transplantation compared with late DTCs.^45,46^ Although more compelling data are needed to elucidate the biological and clinical implications of such observations, one message is clear: given that early detection of tumours may not be sufficient to prevent early dissemination, more efforts focusing on studies of the biological properties of DTCs might hold the key for targeting MRD and halting metastatic relapse from occurring.^52^

### What dictates successful versus unsuccessful metastasis?

The ‘seed and soil’ hypothesis, first proposed by Steven Paget in 1889,^64^ highlighted three key points: first, metastases can only form in specific organs with biologically compatible microenvironments; second, metastases, as well as primary neoplasms, consist of both tumour and host cells; third, metastases result from intimate crosstalk between DTCs (the seeds) and the surrounding milieu (the soil). After more than 130 years, Paget’s pioneering observations still hold true, reinforcing the notion that DTC fate is the result of a fine balance between cell-extrinsic and cell-intrinsic factors^65^ (Fig. 2.6). Specific tumour microenvironments, including seeding in the proximity of blood vessels,^66^ the establishment of premetastatic niches by primary tumours,^67,68^ in part through the release of soluble factors and/or extracellular vesicles^69^ and immune system activity,^70^ at both the primary site^71^ and systemically,^72^ are well-known cell-extrinsic determinants provided by the ‘soil’ that influence metastatic success. Notable cell-intrinsic cues that regulate the ‘seeds’ include genetic and epigenetic plasticity, likely as a consequence of chromosomal instability,^18^ the capacity to self-renew,^4^ proposed to be influenced by epithelial-to-mesenchymal transition (EMT)-inducing transcription factors such as Snail, Twist and ZEB1^73^ and the ability to adapt to foreign sites, for instance via actively remodelling the ECM^74^ (Fig. 2.6). Based on the crucial interplay between different seeds and soils, DTCs can ultimately attain three different destinies: metastatic outgrowth (or colonisation), cell death or tumour dormancy^75^ (Fig. 2.7).

### Metastasis is a highly inefficient, yet lethal process

While further data are eagerly awaited to clarify the ‘when’ and the ‘how’ of DTC dissemination, it is clear that the metastatic cascade is a highly inefficient process^76^ comprising several mechanical and molecular bottlenecks.^77^ Accumulating evidence gathered from experimental mouse models has highlighted several rate-limiting steps within the cascade, which could account for such inefficiency, all of which appear to be linked to post-extravasation events.^66,78^ According to one such study,^63^ which examined the fate of B16F1 melanoma cells following intraportal injection, only a few percentages of extravasated CTCs (1 in 40) were able to form micrometastatic lesions—the rest remaining as solitary cells—and only a small subset of those (1 in 100) eventually progressed into macroscopic tumours—the rest remaining as microscopic lesions.^63^ One of the most important lessons learned from these experiments was the notion that not all DTCs bear the same metastatic potential, perhaps as a reflection of the intratumoural heterogeneity documented in most primary lesions.^79,80^ As a result, only DTCs that are able to give rise to micrometastasis—with a few of those eventually growing into full-blown lesions—are called metastasis-initiating cells.^81^ However, a major hurdle in oncology remains the difficulty in predicting which DTCs will give rise to deadly metastatic relapse. This challenge is attributed to the low frequency of DTCs, the paucity of markers that can unequivocally identify metastasis-prone DTCs and the difficulty in sampling micrometastatic lesions.^33^ Identifying DTC markers and correctly predicting which patients harbour disseminated disease with dangerous potential could lead to better prognostic predictions, reduce unnecessary and often toxic adjuvant treatments and identify targets for a new therapy to prevent metastatic recurrence. Thus, as the capacity for DTC seeding might determine whether a cancer patient lives or dies, it is fundamental to learn more about what shifts the balance between successful versus unsuccessful metastasis.

In the next few sections, we introduce the concept of tumour dormancy and discuss its ties with DTC dissemination in order to highlight how its reversible nature might alter the equilibrium between ‘unsuccessful’ (dormant) and ‘successful’ (growing) metastases, thus possibly dictating the timing of metastatic relapse, or whether relapse occurs at all. We then focus on two key determinants of relapse in HR^+^ breast cancer: the extrinsic effect of targeted therapy, and the consequences of intrinsic HR function modulation.

## Tumour dormancy and reawakening

Tumour dormancy is generally defined as a prolonged state of asymptomatic micrometastatic disease. In cancer of the breast or prostate, cancer cells can remain dormant for years and even decades before recurring as metastatic disease.^23^ During this latent period, patients are considered to be disease-free due to the lack of any symptoms of illness and because they have no detectable neoplasms by clinical imaging.^82^ Often described as one of the most ‘wicked’ cancer cell misbehaviours, tumour dormancy shares many features in common with chronic diseases.^16^ Yet, its nature appears to be reversible, as myriad mechanisms have been shown to induce a switch to reawaken indolent DTCs (see below). Furthermore, tumour dormancy is not exclusively a phenomenon of end-stage tumorigenesis, as it can apply to the presence of occult neoplasms until clinical diagnosis (primary dormancy), and/or to MRD left behind after treatment (metastatic dormancy). Attention, however, must be paid to the molecular underpinnings of these two scenarios as mechanistic differences between primary and metastatic dormancy might exist.^83^

### Cellular and tumour mass dormancy

Two different models of tumour dormancy—cellular and tumour mass dormancy—have been proposed.^75^ Cellular dormancy refers to the presence of solitary or small cell clusters of DTCs that exist in a G0/G1 growth-arrested state^63^ and result from quiescence,^84^ senescence or differentiation.^85^ An inability to properly adhere to the ECM,^86,87^ reduced signalling through the phosphatidylinositol 3-kinase (PI3K)/AKT pathway^88^ and a low ratio of the extracellular signal-regulated kinase (ERK) to the stress-induced kinase p38^89,90^ are some of the plethoras of predominantly cell-intrinsic mechanisms that have been reported to induce cellular dormancy. On the other hand, escape from cellular dormancy has been shown to occur upon increased matrix stiffness through TGFβ1 expression,^91^ following the release of neutrophil extracellular traps (NETs) by inflammatory neutrophils,^92^ and as a result of aberrant activation of the adhesion protein vascular cell adhesion protein 1 (VCAM1) in indolent breast DTCs lodged in the bone marrow via engaging α4β1-expressing osteoclasts.^93^

Tumour mass dormancy refers, instead, to the presence of microclusters of DTCs in which a balance between proliferation and cell death exists so that no net tumour burden is observed.^94^ The two main mechanisms proposed to fine-tune tumour mass dormancy are angiogenesis and immune surveillance, both of which are predominantly cell-extrinsic. Failure to induce angiogenesis is believed to force some DTCs to die, creating an equilibrium between cell death and proliferation.^95,96^ The point at which small masses of dormant tumour cells sense the lack of blood supply and gain the ability to induce angiogenesis is known as the angiogenic switch, which might result in the escape from dormancy and outgrowth of metastases.^97^ Similarly, immune surveillance can keep metastatic tumour cells in check until dormant microclusters of cancer cells are able to evade cytotoxic immunity to give rise to active metastases.^44,98,99^ Importantly, cellular and tumour mass dormancy should not be viewed as separate, static categories—not only because single dormant lesions can transition into a tumour mass dormancy programme,^100^ but also because the biological mechanisms that underlie one model can inform the other.^17^ One example pertains to the angiogenesis regulator thrombospondin, which can be released by non-sprouting endothelial cells to support the quiescence of solitary DTCs,^101^ while also regulating dormancy in small tumour masses with low vascular density.^102^ Nonetheless, whether all the above dormancy scenarios coexist and are functionally relevant in patients remains to be determined.

### Dormancy and its ties with tumour cell dissemination

The first evidence that cancer cells can exist in a dormant state was established following the detection of DTCs in the bone marrow of breast,^103^ colorectal^104^ and prostate^105^ cancer patients without evidence of clinical metastases. It was through advances in immunohistochemistry-based techniques and single-cell profiling that most of such DTCs were in fact found to be in a non-proliferative state, thus alluding to a cellular dormancy model.^106^ Likewise, the presence of non-proliferative CTCs in the blood of clinically disease-free breast cancer patients^107^ is consistent with a tumour-mass dormancy model, as replenishment of CTCs could be indicative of the presence of replicating reservoirs of disease somewhere in the body.^108^ But how does dormancy tie in with DTC spread? With the presumed time needed to acquire a fully malignant phenotype upon departure from the primary tumour, early DTCs are thought to be more likely to undergo dormancy at secondary loci compared with their late counterparts—perhaps as a coping and adaptation strategy to foreign environments.^47^ If this notion holds true, cases of late recurrence—in which a dormancy period presumably occurred—could be attributed to the awakening of early DTCs that remained unscathed during systemic treatment.^46^ Given the ability of primary tumours to fuel growth at secondary sites (as previously mentioned^67^), early DTCs, whilst in a dormant-like state, might contribute to the establishment of premetastatic niches—perhaps through crosstalk with host cells such as endothelial, immune and stromal—thus preparing the soil for late DTCs. Likewise, late DTCs could contribute to relapse and/or foster the awakening of early DTCs via direct and indirect means, thus increasing the risk of metastatic recurrence.^17^ One way this has been hypothesised to occur is through a feedback loop whereby infiltrating cancer cells would educate stromal host cells upon arrival to produce periostin (POSTN), which in turn would increase WNT signalling and foster proliferation of DTCs at secondary sites.^109^ Nevertheless, based on their tight correlation with the poor prognosis of breast cancer patients and their ability to persist under systemic treatment,^110–112^ both DTCs and CTCs are generally considered to be prognostic markers of metastatic relapse. Altogether, this suggests the urgent need to understand the mechanisms governing the reawakening of indolent, metastatic cancer cells.

### Is tumour dormancy the sole explanation for recurrence?

In pondering the mechanisms of metastatic relapse among breast cancer patients, one obvious question is whether early recurrence is simply the consequence of direct metastatic outgrowth, whereas late relapses reflect a period of tumour dormancy. To address this query, it is imperative to consider how long it takes for a single cancer cell to grow into a clinically detectable metastasis. Pioneering measurements of breast tumour volume doubling time (TVDT) carried out by radiographic analysis on more than 800 women concluded that it takes ~12 years on average for a single cell with a 10-µm diameter to reach a clinically detectable mass of 1 cm,^3,43^ and that metastases can have a TVDT up to twofold higher than their matched primary tumours.^33^ However, these initial analyses focused on a small number of samples, without taking into account the vast heterogeneity among breast tumours or the effect that adjuvant therapies might have on their growth rate, as the subjects in this study were untreated.

**Fig. 3:**
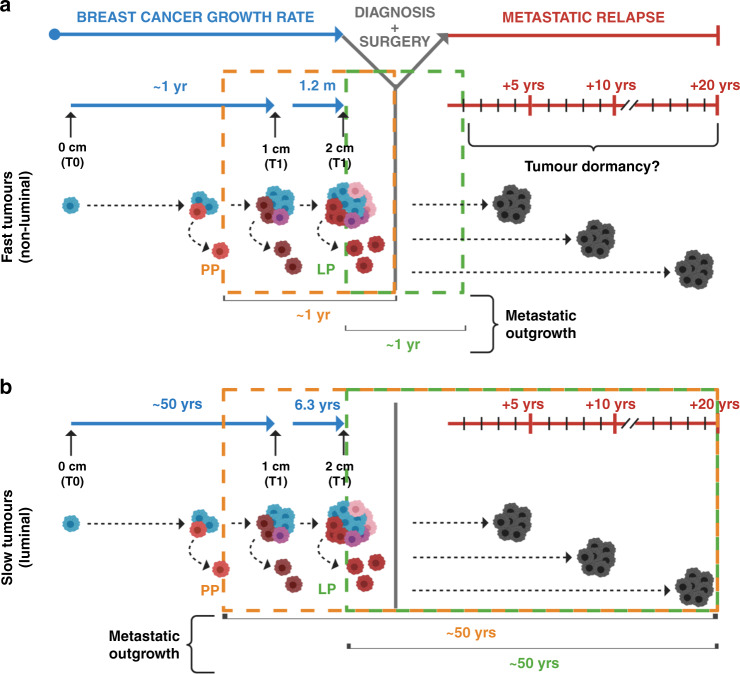
The puzzling timing of metastatic relapse in breast cancer patients. Two different models are shown, based on disparate growth rates observed among breast tumours. **a** The first model is based on fast-growing breast tumours (i.e., non-luminal), which can take ~1 year to give rise to clinically detectable neoplasms (~1 cm in diameter). Assuming comparable proliferative indices for distant lesions, direct metastatic outgrowth of early (parallel progression) or late (linear progression) DTCs could justify cases of early metastatic recurrence. Alternatively, tumour dormancy might be responsible for late relapses of fast-proliferating tumours. **b** The second model is based on slow-growing breast tumours (i.e., luminal), which can take up to 50 years to become clinically detectable. Assuming comparable proliferative indices for distant lesions, direct metastatic outgrowth of DTCs without dormancy could explain both early and late cases of recurrence, regardless of the timing of DTC spread. DTC disseminated tumour cell, LP linear progression, PP parallel progression, T tumour stage, YRS years. Figure created with BioRender.com.

A more extensive study^113^ on ~400,000 patients from Norway revealed striking variation between breast tumours in the time taken for a lesion to double from 1 to 2 cm, ranging from less than 1.2 months (for the 5% fastest-growing cancers) to 6.3 years (for the 5% slowest-growing tumours).^113^ Assuming constant exponential or logarithmic growth and comparable proliferative indices between primary and metastatic tumours,^33^ this analysis would now suggest a range of ~1 to more than 50 years for a tumour to reach a clinically visible size^83^ (Fig. 3). This model is also consistent with ultrasonography studies reporting disparate TVDTs between the different molecular subtypes of the disease.^114,115^ Thus, if breast cancers have such disparate growth rates, it might be more plausible to conceive a period of dormancy for fast-growing, non-luminal tumours, in cases when metastatic relapse occurs later than the first few years from surgery (Fig. 3a). By contrast, a period of dormancy would now seem redundant for luminal lesions, the low proliferative index of which might sufficiently justify cases of metastatic relapse up to 20 years after diagnosis^83^ (Fig. 3b).

Although exciting new data have revealed the identity and functional role of several dormancy-related mechanisms in different model systems, questions still remain as to how often dormancy is a factor in metastatic recurrences for breast cancer, and whether it can be exploited clinically to prevent relapse. Better knowledge of tumour growth patterns in metastatic sites, and of the status of DTCs in their natural metastatic setting (see the section on rapid autopsy studies), is needed to determine which DTC features might contribute to lethal metastasis.

### Dormancy-permissive sites and the timing of metastatic relapse in breast cancer

The most well-characterised dormancy-permissive site of the body is the bone marrow,^100^ largely due to the fact that it is technically easier to sample than other sites. Here, three niches that might actively support dormancy—presumably due to the lack of factors that promote reawakening—have been identified: stem cell, immune and vascular niches (reviewed elsewhere^17^). In this view, the aforementioned wide window of metastatic relapse observed among breast cancer patients can perhaps be partially explained by the unique metastatic tropism displayed by the different molecular subgroups of the disease.^13^ Accordingly, the shorter survival time observed among patients with non-luminal tumours might be linked to the propensity of the latter to disseminate to visceral organs—including the brain, previously mentioned as the deadliest among all metastatic sites^15^—as opposed to the longer prognosis showed by luminal patients whose tumours appear to preferentially recur to the bone.^28,116^ A deeper understanding of cell-extrinsic and cell-intrinsic factors in each of these metastatic milieus could perhaps help to unveil whether organ tropism, together with the timing of DTC spread, is instructive for dormancy and, therefore, might explain the wide window of relapse that plagues breast cancer patients.

## Key determinants of relapse in HR^+^ breast cancer

### The extrinsic effect of targeted therapy

**Fig. 4:**
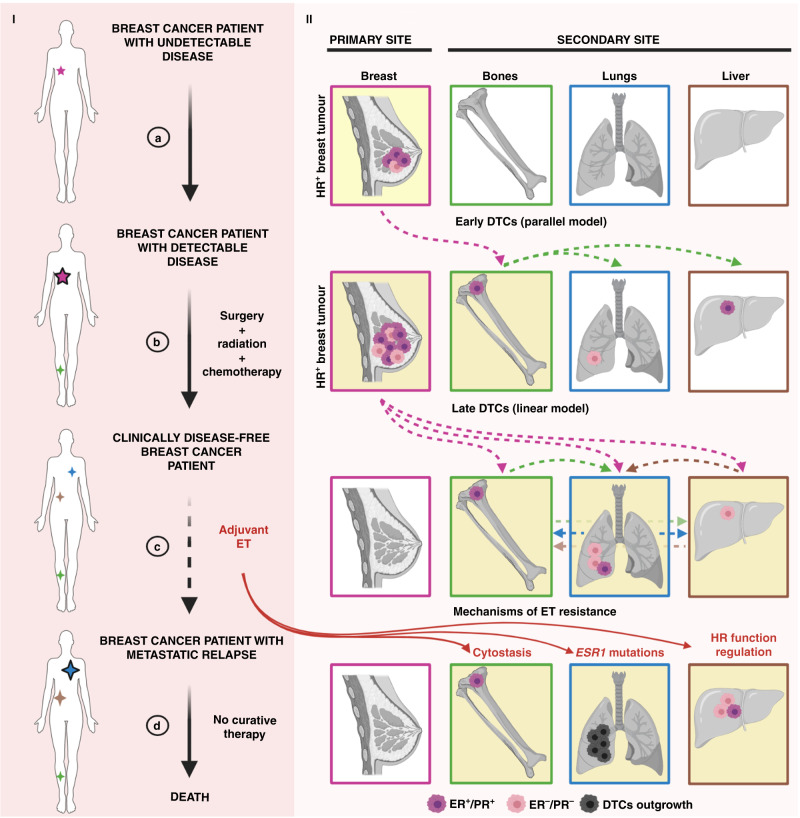
The effects of cell-extrinsic and cell-intrinsic determinants in dictating breast cancer outcomes. Part I The journey of a breast cancer patient from the development of undetectable disease and its clinical discovery (**a**), through its surgical removal (**b**) and adjuvant ET (**c**), to metastatic relapse and death (**d**). The presence of tumour lesions across the body is indicated by stars—the smaller referring to the clinically undetectable ones (no black borders), the bigger ones to the clinically detectable ones (black borders). Part II The development of an HR^+^ breast tumour lesion in the breast (primary site), comprising a mixture of ER^+^/PR^+^ and ER^–^/PR^–^ cells (**a**). DTC escape from the primary site can occur early (parallel model, **a**) and/or late (linear model, **b**) during tumorigenesis (dotted pink lines), although the HR phenotype of DTCs at these stages is often unclear. Bones, lungs and liver are represented as common secondary sites for breast cancer metastases, albeit the sequential patterns of DTC spread among these organs are still elusive (green, brown and blue dotted lines). Targeted treatment for HR^+^ breast cancer patients relies on adjuvant ET. Several mechanisms of ET resistance (**d**)—cytostasis, *ESR1* mutations and HR function regulation—contribute to DTC outgrowth. DTC disseminated tumour cell, ER oestrogen receptor, ET endocrine therapy, HR hormone receptor, PR progesterone receptor. Figure created with BioRender.com.

In addition to surgery, radiation and chemotherapy, which are the mainstays of treatment for breast tumours with a reasonable risk of recurrence,^117^ HR^+^ breast cancer patients can additionally benefit from endocrine therapy (ET)—including selective oestrogen receptor modulators (e.g., tamoxifen), selective oestrogen receptor degraders (e.g., fulvestrant) and/or aromatase inhibitors (e.g., letrozole)^118^ (Fig. 4, part I). Since the discovery of ER as a predictive biomarker of response to ET,^9^ the use of hormone therapy has significantly reduced breast-cancer-related mortality.^119^ However, despite being administered in the adjuvant setting to eliminate MRD and prevent metastatic relapse (Fig. 4, part Ic), up to 30% of HR^+^ tumours eventually develop mechanisms of resistance to ET.^120^ In addition, accumulating evidence has suggested that ET largely induces cytostatic effects, blocking proliferation and forcing cells to enter a dormancy state, rather than killing DTCs^121–124^ (Fig. 4, part IId). Although it is unclear whether this applies to all HR^+^ breast cancers and all ET agents, one possibility is that the long latency period experienced by many HR^+^ patients prior to recurrence is partially mediated by targeted therapy enforcing a dormant state, until ET-resistant DTCs eventually emerge and give rise to deadly metastatic disease (Fig. 4, part Id). As our understanding of the effects of ET on HR^+^ breast cancers is largely restricted to the use of actively growing tumours or cell lines, there is an urgent need for more clinically relevant models (such as rapid autopsy studies, described below) to reveal how DTCs respond to hormone therapy, or to other targeted treatments (e.g., HER2-directed therapy for HER2^+^ breast cancers). In addition, more efforts should be focused on the identification of new biomarkers and molecular targets for the treatment of HR^+^ therapy-resistant tumours.^125^

A new therapeutic standard for metastatic HR^+^ breast cancer patients is evident in the development and approval of cyclin-dependent kinase (CDK)4/6 inhibitors—palpociclib, ribociclib and abemaciclib—administered in combination with ET.^126^ By blocking the function of the CDK4/6–cyclin D1 complex, CDK4/6 inhibitors prevent cell-cycle progression from G1 to S phase, thereby halting cancer cell proliferation.^127^ Thus, in line with cyclin D1 amplification as a common event in luminal tumours,^128^ and the known role of ER signalling as an activator of the CDK4/6–cyclin D1 complex,^129^ CDK4/6 inhibitors have been shown—in combination with ET—to significantly extend the progression-free survival (PFS) of metastatic HR^+^ breast cancer patients over ET alone. Accordingly, the combined use of CDK4/6 inhibitors and adjuvant ET has been approved by the FDA for the treatment of metastatic HR^+^ breast cancer patients.^130^ Nonetheless, additional results from upcoming clinical trials^126^ are eagerly awaited to determine: first, the presence of predictive biomarkers (besides ER positivity); second, the optimal sequential management of metastatic HR^+^ breast cancer patients with early- and late-stage diseases; third, any putative cytostatic effects these drugs might have on DTCs if administered in the adjuvant setting (prior to clinical diagnosis of metastasis). Interestingly, in a study by Asghar et al.,^131^ cell-cycle dynamics were shown to determine the response to CDK4/6 inhibitors. That is, cancer cells exiting mitosis with low levels of CDK2 (quiescent cells) would strictly depend upon CDK4/6 function to re-enter the cell cycle, whereas cells exiting mitosis with high levels of CDK2 (proliferating cells) would bypass the requirement for CDK4/6 activity.^131^

### The intrinsic effect of HR function regulation

Given the dependence of tumour cells on oncogenic signalling for survival,^132^ the modulation of oncogenic pathways might play an important role in shaping the fate of DTCs at distant sites. In the context of HR^+^ breast cancer, ER and PR are master regulators of breast cancer cell proliferation and survival.^133^ Alterations in the expression and/or function of HR are often noted upon metastatic recurrence. Typically, but not always, such alterations are associated with progression on ET therapy. Multiple independent studies have in fact reported significant HR discordance rates between primary breast tumours and matched metastatic lesions^134–140^ (Fig. 4, part II), highlighting several issues. First, DTCs do not always share the same phenotypic characteristics of their corresponding HR^+^ primary tumours. Second, PR is more frequently altered than ER. Third, the conversion status of ER from positive into negative tends to correlate with worse overall survival of patients. Finally, adjuvant ET can significantly affect HR expression. In light of the molecular heterogeneity amongst luminal tumours,^22^ and the reduction in the cut-off value for defining ER^+^ cancers from 10% to 1% of positively stained cells,^141^ the fact that women with HR^+^ tumours present with ER^–^ DTCs is perhaps not much of a surprise, per se. However, this finding might be a sign of increased fitness displayed by ER^–^ DTCs in metastatic sites. Of particular relevance in the oestrogen-deprived milieu of clinically disease-free HR^+^ patients, the selection of ER^–^ DTCs at distant loci could derive from three scenarios, which are not mutually exclusive: selective ET-induced cell death of ER^+^ DTCs,^118^ loss of ER in formerly ER^+^ DTCs upon oestrogen deprivation (HR function regulation)^142^ or increased metastatic potential of pre-existing ER^–^ DTCs.^143^ Although none of these scenarios can be ruled out with existing data, it is clear that DTCs persist in many HR^+^ patients even after standard ET, contributing to an increased risk of relapse in the long term (Fig. 4). Unfortunately, it is not currently possible to discern which patients present with clinically significant versus insignificant MRD (see the section on early detection of recurrence, described below), nor to assess the phenotypic properties of DTCs in various tissues to optimise guidance of systemic therapies. Therefore, this represents another area in dire need of more research.

## Rapid autopsy studies as an opportunity to better understand DTCs

Rapid autopsy studies—the post-mortem collection and examination of tissues from cancer patients (Box 1)—can provide precious specimens for the analysis of DTCs in sites other than the bone marrow.^144^ Indeed, autopsy research is what informed Stephen Paget’s hypothesis, as he was struck by the observation of frequent metastases in relatively poorly vascularised organs after scrutinising more than 900 autopsy records.^12^ As well as providing information on the non-random pattern of metastatic dissemination, rapid autopsies allow more extensive multiregional sampling and, therefore, a more comprehensive evaluation of tumour evolution. Notwithstanding the intrinsic caveats embedded in comparative studies between primary and metastatic sites,^35^ the results from most autopsy studies have revealed a high degree of genetic and phenotypic divergence among paired lesions,^37,145,146^ strengthening the notion that diverse metastasis-prone cancer cells can precociously break away from primary tumours. This implies that metastatic traits might be intrinsically embedded within some incipient primary neoplasms, in line with the parallel model^33^ and the concept that some tumours are ‘born to be bad’.^147^ However, although early metastatic dissemination does not always appear to be an acquired process, the opposite might hold true for metastatic outgrowth of early DTCs at distant sites—presumably needing more time to acquire further mutations in order to give rise to active metastases—thus accounting for the acquisition of unique alterations only displayed by metastatic lesions.^35,47^ More importantly, autopsy studies offer the unique opportunity to assess DTCs in their natural metastatic niches, as opposed to using methods that rely on aspirating DTCs from the bone marrow, for example. This approach provides an extraordinary opportunity not only to understand why certain patients experience relapse and others never do so, but also to examine all disease reservoirs within the same individual in order to unveil the occult behaviour of some lesions versus the re-emergence of others. Lastly, through the creation of autopsy-patient-derived models, rapid autopsies of cancer patients can facilitate the testing of new pharmacological agents and identification of actionable targets using metastatic tumours as starting material^144^ (Box 1). Thus, it is hoped that rapid autopsy programmes will soon be implemented as routine clinical platforms to support cancer research and address outstanding issues, including the pathogenesis of metastatic relapse in breast cancer patients.

Box 1 How rapid autopsy studies can inform on metastatic dissemination and relapse **Definitions**

- Rapid autopsy: rapid post-mortem collection, examination and biobanking of tissues—fresh, snap-frozen and fixed—from deceased patients shortly after death.
- Rapid autopsy cancer programme: coordinated effort among oncologists, pathologists and scientists aimed at collecting specimens from cancer patients within a post-mortem interval (PMI) of 6–8 h before key biological information within the tissues of interest is lost.

**Advantages**

- Multiregional biopsies: to conduct extensive, spatial sampling of tissues—primary and metastatic, cancerous and normal—for in-depth, high-resolution multi-omics (i.e., genomic, transcriptomic and proteomic) analysis.
- Physiological model: to analyse DTCs in their natural metastatic niche(s).
- Source for unique model systems: to generate novel, ex vivo living patient-derived models—autopsy-derived xenografts (ADXs) and organoids (ADOs)—of metastatic tumours from sites that would otherwise be difficult to sample for functional evaluation (i.e., to unveil new predictive biomarkers of drug response and test novel therapeutics).
- Cancer evolution: to study the phylogenetic relationship of each sampled site to each other and infer the complete clonal evolution of a neoplasm.
- Dormancy: to examine why some DTCs lodged in certain organs of the human body (e.g., bone marrow) become dormant for years to decades.
- Drug resistance: to study why DTC spread across different sites responds differently to therapy, with some developing resistance and others remaining sensitive to treatment.
- Recurrence: to understand why only some DTCs residing in certain sites of the human body give rise to active metastases, ultimately responsible for patient’s relapse.

**Ultimate goal**

- To generate new hypotheses on how to better tackle MRD and prevent metastatic recurrence. *DTC* disseminated tumour cell, *MRD* minimal residual disease.

## Can earlier detection of recurrence improve breast cancer outcomes?

The risk of metastatic relapse weighs heavily on the minds of patients, physicians and caregivers for years, sometimes decades, after treatment of the primary tumour is complete. Nearly 17 million cancer survivors are living in the United States, 3.9 million of whom are breast cancer survivors,^148^ and repeated monitoring for cancer recurrence in these individuals presents a significant challenge to healthcare delivery systems. For breast cancer patients, current American Society of Clinical Oncology (ASCO) and National Comprehensive Cancer Network (NCCN) guidelines limit follow-up care to mammography, medical history and physical exam, stating that ‘in the absence of clinical signs and symptoms suggestive of recurrent disease, there is no indication for laboratory or imaging studies for metastases screening’.^149,150^ Despite these guidelines, however, many patients receive high-cost imaging analysis (CT, brain or body MRI, PET and bone scans) and tumour marker blood tests during routine follow-up exams, exposing them to radiation and increasing healthcare costs.^151–154^ So, what has led to the current precarious balance between the desire to detect recurrence early and clinical guidelines that limit the use of diagnostic tests?

Several large studies and clinical trials published between 1994 and 2007 are often cited as evidence against the use of more intensive monitoring for recurrence.^155,156^ These studies compared the current (at the time) clinical follow-up regimen with various more intensive regimens that included chest X-ray, bone scans, liver ultrasonography and blood tests measuring haemoglobin, white blood cell count, platelet count, calcium, sedimentation rate, liver enzymes (alkaline phosphatase and alanine aminotransferase) and the tumour marker CA15-3. Although some of these more intensive studies reported earlier detection of metastatic relapse,^157^ they found no improvement in disease-free or overall survival (OS) compared with the standard clinical follow-up regimen. Several of these studies noted that more intensive follow-up regimens were resource-intensive and resulted in more than double the healthcare costs.^155^ By contrast, a 2009 meta-analysis^158^ found that, when locoregional recurrence was detected by routine mammography before patients had symptoms, their OS was better than when relapse was detected by the patient themselves. This study concluded that the earlier detection of all breast cancer recurrences would result in an absolute reduction in mortality of 17–28%.^158^ A major difference between these studies is that modern mammography has been optimised for the sensitive early detection of tumours, whereas the imaging and biomarkers used in the earlier analyses might have been less sensitive. Indeed, none of the studies that informed the current guidelines used modern sensitive imaging modalities (e.g., CT, PET or MRI). Additionally, these studies were conducted before targeted therapies—currently used to treat metastatic breast cancer and proven to significantly reduce breast-cancer-related mortality^8^—were widely available. By contrast, current imaging modalities are capable of detecting small metastatic lesions in lymph nodes,^159^ and every year, new targeted therapies extend PFS in metastatic breast cancer patients.^160^

The development over the past decade of non-invasive biomarker assays promises to enable the low-cost early detection of cancer.^161^ A myriad of non-invasive biomarker assays and analytes is currently being investigated, including the detection of circulating tumour DNA (ctDNA) based on cancer-specific mutations and DNA methylation, CTCs, tumour-derived extracellular vesicles, circulating RNAs, tumour-associated proteins and tumour-educated blood platelets (reviewed in detail elsewhere^162,163^) Several of these assays have demonstrated success in the early detection of breast cancer recurrence. In two studies published in 2019, ctDNA has detected a median of 8.9 and 10.7 months prior to clinically detectable disease recurrence, with sensitivities of 89% and 96%, respectively.^164,165^ This window of lead time in which ctDNA is detectable in blood, but metastatic recurrence is not yet visible on imaging, provides an exciting opportunity to treat the disease during the dormancy phase (see the section below on the different strategies that could be used to tackle MRD, Table 1).

**Table 1:** Exploiting tumour dormancy as a window of therapeutic opportunity to target MRD.

Potential strategy	Actionable mechanism(s)	Caveat(s)
1. Prevent dormancy	Early diagnosis and removal of primary tumors	Early dissemination of DTCs (parallel model of metastasis) Intrinsic or acquired resistance to therapies
	To block tumour cell dissemination to secondary sites	
	Differentiation therapies	
2. Reverse dormancy	Angiogenic factors + conventional anti-proliferative therapies	Intrinsic or acquired resistance to therapies
3. Prolong dormancy	Immunotherapies	Immunoediting (escape)
	Anti-angiogenic therapies	Intrinsic or acquired resistance to therapies
	Dormancy-maintaining therapies (e.g., cytostatic agents)	
4. Eradicate dormancy	Immunotherapies	Immunoediting (escape)
	Differentiation therapies	Intrinsic or acquired resistance to therapies
	To block tumour cells Achilles heels (e.g., induce apoptosis, block survival or alter metabolism pathways)	

Although the concept of measuring and treating MRD is routine in the management of haematological malignancies,^166^ it is a relatively new idea in breast cancer treatment, thus raising concerns about the timing and selection of appropriate therapy, as well as the potential to overtreat MRD that might never otherwise manifest clinically. Nonetheless, hormone and chemotherapy administered after treatment of primary breast tumours have been shown to improve survival,^8^ presumably by eradicating undetectable MRD. As previously discussed, metastatic tumours can differ from the primary tumour in both mutation spectrum and subtype, which might contribute to therapy resistance. Many of the novel biomarker assays developed in the past decade present an exciting opportunity for the so-called ‘liquid biopsy’ detection of these molecular changes in metastatic recurrence through non-invasive measurements of body fluid.^161^ Blood-based detection of mutations in *ESR1* (the gene that encodes ER1) is associated with resistance to hormone therapy, and in *PIK3CA*, is associated with sensitivity to CDK4/6 inhibitors.^167,168^ These results demonstrate that non-invasive tests might not only identify recurrence early, but also inform the selection of optimal treatment strategies.

In view of advances in imaging and screening technologies, perhaps now is a suitable time to revisit clinical guidelines and determine if new follow-up regimens that use non-invasive biomarker assays, potentially followed by imaging in the case of positive biomarkers, can detect metastatic recurrence early, and whether administering new targeted therapies at these earlier timepoints might improve survival. ASCO last updated its recommendations for the use of tumour markers in breast cancer in 2007, and concluded that there was no evidence that detecting and treating early metastatic findings using the tumour markers available at the time (CA15-3, CA-27.29 and CEA) impacted patient outcomes.^19^ It will require a significant investment of resources, and high-risk tolerance, to design and implement clinical trials that challenge current follow-up guidelines, which are based on extensive research from the previous decade, indicating that the early detection and treatment of metastasis do not improve survival. However, our current understanding of metastatic dormancy, metastatic evolution, acquired resistance and metastatic niches suggests that detecting and treating recurrence earlier might be our best opportunity to improve patient outcomes.

## Conclusions and future directions

Despite metastatic disease being the main cause of death in cancer patients, knowledge about the biology of this lethal process is lacking. However, with advances in genomic sequencing technologies and accumulating data in favour of the predominance of the parallel model over the linear model of dissemination, four important lessons have been learned. First, in most patients who will experience recurrence, metastases are already present at the time of diagnosis. Second, despite their genomic similarity to the trunk of the tumour’s evolutionary tree, metastases are distinct biological entities, containing important phenotypic differences compared with their primary source. Third, metastases, as well as primary neoplasms, are subject to evolution dictated by pharmacological pressure, tissue-specific environments and cellular plasticity. Finally, some micrometastases might exist in a non-proliferative, dormant-like state for months to decades.

Altogether, these data have significant clinical implications. First, attempts to prevent initial metastatic dissemination might hold little therapeutic benefit—even though early detection and treatment of most primary breast tumours is curative, tumours that are ‘born to be bad’ probably disseminate prior to diagnosis. Next, caution must be taken when using primary tumours as a proxy for clinical decisions on systemic therapies to prevent or treat metastatic disease, unless little divergence between paired lesions is observed. In this respect, non-invasive liquid biopsy biomarker assays could help to identify molecular changes in metastases. In addition, an urgent need exists to assess the phenotypic properties of DTCs in their native states, in order to understand how some DTCs, but not others, contribute to metastatic recurrence. Finally, it might be possible to exploit tumour dormancy in some, but probably not all, cases as a window of opportunity to tackle MRD and/or prevent metastatic relapse (Table 1).

Future directions should be centred on identifying dangerous versus indolent DTCs and finding new DTC drug targets, while also using modern imaging and biomarker assays to accurately determine who needs such therapy, in order to avoid overtreatment. Importantly, all of the above strategies—prevent, reverse, prolong and eradicate dormancy (Table 1)—will require the development and use of more clinically relevant models and human samples, as well as clinical trials of new ‘intensive’ follow-up monitoring programmes. With progress in these areas, it is hoped that new knowledge will converge in the near future to prevent recurrences and deaths from cancer.

## Data Availability

Not available.
